# Relationship of Starch Pasting Properties and Dough Rheology, and the Role of Starch in Determining Quality of Short Biscuit

**DOI:** 10.3389/fpls.2022.829229

**Published:** 2022-03-28

**Authors:** Liang Liu, Tao Yang, Jianting Yang, Qin Zhou, Xiao Wang, Jian Cai, Mei Huang, Tingbo Dai, Weixing Cao, Dong Jiang

**Affiliations:** College of Agriculture, Nanjing Agricultural University, Nanjing, China

**Keywords:** gelatinization, quality, short biscuit, **s**tarch, wheat

## Abstract

Starch plays an important role in food industry. In this study, three wheat cultivars with different protein contents were used to investigate the different ratios of starch addition on starch pasting properties, starch thermal performance, dough rheology, biscuit quality, and their relationships. Results showed that with the increase in starch content, gluten, protein and glutenin macropolymer (GMP), lactic acid solvent retention capacity (SRC), sucrose SRC, and onset temperature (To) decreased, while most pasting parameters and gelatinization enthalpy (ΔH) increased. Viscosity parameters were significantly negatively correlated with dough stability time, farinograph quality number (FQN), and sucrose SRC. Biscuit quality was improved by starch addition, indicated by lower thickness and hardness, higher diameter, spread ratio, and sensory score. Viscosity parameters were positively correlated to diameter, spread ratio, and sensory score of biscuit, while negatively correlated to hardness and thickness of biscuit. Image analysis showed that the crumbs of biscuit were improved as shown by bigger pores in the bottom side. The results provide useful information for the clarification of the role of starch in determining biscuit quality and the inter-relationships of flour, dough, and biscuit.

## Introduction

Biscuits are one of the most popular wheat products due to their ready to-eat, long shelf-time, and wide-variety (Moriano et al., 2018). The biscuit production had increased from 1.05 to 12.5 million tons with an annual growth rate of 18.0% from 2004 to 2019 in China (Yang et al., 2022). Wheat flour with low content of protein and gluten is believed an ideal material for biscuits, cookies, and other bakery foods (Manley, 2011). However, most of the commercial wheat grains in China had medium to high protein content due to higher N fertilizer input. Xu et al. (2016) analyzed 7,561 samples of 742 varieties from the main production region of wheat in China from 2006 to 2015 and found that the ratio of weak gluten wheat was lower than 1%. It is thus of importance to produce wheat flour with low-protein content for the biscuit industry in China.

It is believed that biscuit quality is closely related to flour protein content. The hardness of biscuits are suggested to increase gradually with elevated protein and gluten levels (Fustier et al., 2008; Pauly et al., 2013). Good quality soft wheat flour produces large spread cookies with a large diameter and low thickness. However, Moiraghi et al. (2011) found that protein and gluten contents were not related to cookie diameter. The protein content in flour for biscuit baking varied greatly among different flours. In the U.S., the protein content of five popular brands of self-rising flours applied in biscuit-baking varied widely, with protein contents of 6.7, 8.5, 9.4, 9.7, and 10.0%, respectively (Ma and Baik, 2018). The wide variation in the protein content of commercial self-rising flours indicates that flour protein content may not be a critical wheat characteristic for biscuit production (Ma and Baik, 2018). During the dough formation, gluten proteins in the flour are hydrated to form gluten networks. The gluten network is important in bread and other soft products, whereas it does not play a fundamental role in biscuits (Schober et al., 2003). Actually, the gluten network has to be only slightly developed to obtain a cohesive but not a very elastic dough. It is also reported that biscuits can be produced without gluten. Reasonable textural quality biscuits can be made from flours of many different types of gluten-free grains, including sorghum, pseudocereals, and legumes to meet the demand of population affected by the celiac disease (Di Cairano et al., 2020; Adedara and Taylor, 2021). So gluten may play a secondary role in the production and end-product quality of biscuit (Engleson and Atwell, 2008).

Although starch is the most abundant component of wheat grain (about 70–75%), its role in biscuit baking has not been paid enough attention. Our previous studies showed that the characteristics of starch are strongly related to biscuit quality (Zhou et al., 2018; Yang et al., 2022). The texture of biscuits does not depend on protein/starch structure, but primarily on starch gelatinization and super-cooled sugars (Thejasri et al., 2017). Adedara and Taylor (2021) reported that the increased proportion of pre-gelatinized flour starch in the dough reduced the breaking strength of biscuits. Ma and Baik (2018) reported that biscuit-specific volume exhibited positive correlations with the peak viscosity of starch. In biscuit structure, gas cells with various sizes and shapes are embedded in the matrix of gelatinized starch, fat, and sugar. The gelatinization of starch contributes to the formation of the biscuit matrix (Pauly et al., 2013).

Weak gluten wheat was less supplied because it is usually associated with low yield due to low N input. Starch addition is an effective way to produce flour with low protein and gluten content to meet the requirement for biscuit baking. However, the functionality of starch on the processing quality during biscuit making is far less understood than protein. In this work, recombined flour of different starch gradients were produced to probe and clarify the influence of different incorporation levels of starch addition on starch pasting properties, starch thermal performance, dough rheology, biscuit quality, and their inter-relationships. This work will be attempted to disclose how starch addition may regulate the properties of flour, dough, and biscuits and provide guidance for the improvement in biscuit quality.

## Materials and Methods

### Materials

Three widely grown winter wheat cultivars (*Triticum aestivum* L.) in Jiangsu province and surrounding areas were taken in the present experiment. The three cultivars contain different grain protein content (GPC), such as Ningmai 13 (NM13, low GPC), Yangmai 16 (YM16, medium GPC), and Zhengmai 9023 (ZM9023, high GPC). Wheat grain was tempered to 14% moisture prior to milling for 12 h with a laboratory Miller (ZS70-II, grain and oil foodstuff machine factory, Zhuozhou, China). The flour yield was about 70%. The starch contents of NM13, YM16, and ZM9023 were 78.81, 78.30, and 77.61%, respectively.

### Preparation of Flour Varied With Starch Content

Starch from flour of each cultivar of wheat was isolated with the method of Gujral et al. (2013). Briefly, wheat flour was mixed with a moderate quantity of water to form dough. The dough was washed thoroughly with 0.2 M NaCl solution. The slurry was filtrated through a sieve, followed by being centrifugation at 3,000g for 10 min. After isolation, the purified starch was freeze-dried using an Alpha 1-4 LD plus freeze dryer (Christ, Germany). The purified starch obtained from the above-mentioned process was returned to the native flour of the corresponding cultivars to obtain flour with different starch contents. For each cultivar, the additive amount of starch was set at 5, 10, 15, 20, and 25 g, respectively, to make a final amount of 100 g for each recombined flour. The samples without addition of starch were used as control (0). Three biological replicates were used for further analysis.

### Contents of Protein, Gluten, Glutenin Macropolymer, and Starch Components

Flour N content was determined using the micro-Kjeldahl distillation method of AACC 46-11A (2000), and the protein content was calculated as N content multiplied by 5.7. Gluten content was determined according to AACC 38-12.02 procedure (AACC, 2000) with a gluten instrument (Perten instruments AB, Stockholm, Sweden). The GMP content was determined by the method described by Weegels et al. (1994). Briefly, 50 mg of flour sample was suspended in 1 ml of SDS (1.5%) solution and then centrifuged at 15,500 g at 20°C for 30 min. The sediment was washed twice with SDS solution (1.5%). Then the sediment was dissolved in 2 ml NaOH (0.2%) for 30 min, and the N content in the sediment was recorded as GMP content. Contents of amylose and amylopectin were determined using dual-wavelength spectrophotometric assay following the method of Zhang et al. (2010).

### Solvent Retention Capacity

Solvent retention capacity tests of flour were determined according to AACC 56-11 (AACC, 2000). Briefly, SRC is the weight of solvent held by flour after centrifugation. It is expressed as a percent of flour weight. Four solvents are independently used to produce four SRC values: water SRC, 50% sucrose SRC, 5% sodium carbonate SRC, and 5% lactic acid SRC.

### Pasting and Thermal Properties

Pasting properties were analyzed with a Rapid Viscosity Analyzer 130 (RVA-3D super-type, Newport Scientific, Australia) according to AACC 76-21 (2000). Thermal properties were measured by differential scanning calorimetry 8,000 (DSC) (PerkinElmer, USA) according to the method described in our previous study (Yang et al., 2022). The gelatinization temperature (To, onset temperature; Tp, peak of gelatinization temperature; Tc, conclusion temperature) and gelatinization enthalpy (ΔH) were calculated by Pyris software.

### Rheology and Texture Profile of Dough

Dough rheology was determined using Brabender Farinograph-E (Duisburg, Germany) following the method of Chinese national standards GB/T 14614-2006 (Committee, 2006). Dough texture profile (adhesiveness and cohesiveness) was determined by the texture analyzer (TA. XT2i, Stable Micro Systems, Surrey, United Kingdom) using a 25 mm Perspex cylinder probe (P/25P) with 5-kg load cell. The conditions for TPA were kept at: pre-test speed of 0.5 mm/s, test speed of 0.5 mm/s, post-test speed of 10 mm/s with a force of 40 g.

### Biscuit-Making Procedure

The biscuit-baking procedure was carried out according to the Commercial Industry Standard SB/T10141-93 (China, 1993). The formula included flour (300 g, 14% moisture basis), sugar (85.5 g), maltose (13.8 g), shortening (45 g), cream(6 g), sodium chloride (0.9 g), sodium bicarbonate (0.21 g), ammonium bicarbonate (0.9 g), non-fat dry milk(13.8 g), and egg (50 g). The dough was developed using a Hobart N5 mixer (Hobart Corporation, Troy, OH, United States) and then sheeted to a thickness of 2.5–3 mm, followed by being cut using a rotary mold with 40 mm in diameter, and finally being baked at 200°C for 10 min. Biscuits were cooled for 30 min after removing from the oven and then the baking-quality-related parameters were analyzed.

### Biscuit Quality Test

Width, thickness, and spread ratio of biscuit were measured according to the method of Kaur et al. (2015). Biscuit width (W) was measured by laying six biscuits edge to edge and rotating the biscuits 90° and rearranging them to get the average width. Biscuit thickness (T) was measured by stacking six biscuits on top of each other and restacking them in different orders to get the average thickness. The spread ratio (SR) was calculated as follows: SR = W/T.

The color of biscuit was determined using a Chroma Meter (CS-10, Caipu company, Hangzhou, China). Five replicates of each biscuit type were measured from five different points. The color parameters determined were L* (0, black; 100, white), a* (−100, green; + 100, red), and b* (−100, blue; + 100, yellow).

Biscuit hardness was determined by a texture analyzer (TA. XT, Stable Micro Systems, Surrey, United Kingdom) using a sharping P/5 probe according to our previous method (Zhou et al., 2018).

Image analysis of the bottom side of the biscuit was carried out according to Wilderjans et al. (2008). The biscuits were placed on a flatbed scanner (Epson Perfection V30, Seiko Epson Corporation, Japan), and images of the bottom side of the biscuit were captured. The images were processed using Image J version 1.49 software (NIH, Bethesda, United States), and two features, namely, mean cell area and cell to total area ratio were selected to reflect the collapse condition of the biscuits. The bottom side cells detection was conducted on the binary images based on the Otsu thresholding algorithm (Ohtsu, 1979).

Sensory evaluation of biscuits was evaluated by a panel of ten trained judges from the laboratory according to the method of Commercial Industry Standard SB/T10141-93 (China, 1993). Biscuits were coded with different numbers and presented to the evaluator at a random order to evaluate the appearance, mouth feel, texture, crispness, and general acceptability.

### Statistical Analysis

All data were subjected to one-way ANOVA using the SPSS Version 10.0. ANOVA mean comparisons were performed in terms of the least significant difference (LSD), at the significance level of *p* < 0.05. Correlation regression was analyzed using Sigmaplot 12.5. All the tests were performed with three technical repetitions.

## Results

### Flour Protein, Glutenin Macropolymer, Wet Gluten, Damaged Starch, and Starch Components

**FIGURE 1:**
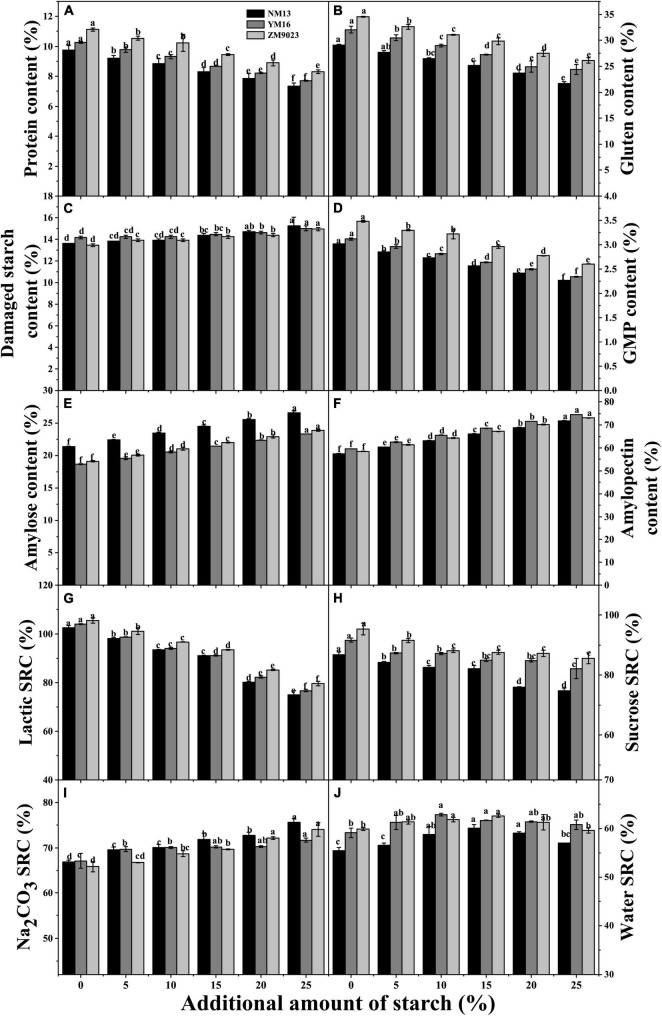
Effects of the addition amount of starch on contents of protein **(A)**, gluten **(B)**, GMP **(D)**, damaged starch **(C)**, starch components (**E**, amylose content; **F** amylopection content) and SRC in the recombined flour (**G**, Latic SRC; **H**, Sucrose SRC; **I**, Na_2_CO_3_ SRC; **J**, Water SRC). NM13, YM16, and ZM9023 indicate Ningmai 13, Yangmai 16, and Zhengmai 9023, respectively.

Protein content in native flour was significantly different among the three cultivars (Figure 1A), and that of NM 13, YM 16, and ZM 9023 was 10.7, 11.25, and 12.2%, respectively. The addition of starch linearly decreased protein content in the recombined flour. The final protein content in the recombined flour still followed the pattern as in native flour among three cultivars. Increasing starch addition up to15%, the corresponding protein content decreased to 9.1, 9.5, and 10.4% of NM 13, YM 16, and ZM 9023, respectively. Consistent with protein, contents of gluten and GMP in the recombined flours also linearly decreased with the increase of starch addition in the three cultivars (Figures 1B,D), and at the same starch addition, ZM 9023 had the highest value, and NM 13 the lowest. The addition of starch linearly increased contents of damaged starch, amylose, and amylopectin in the recombined flours (Figures 1C,E,F).

### Solvent Retention Capacity

Lactic acid SRC (LASRC) and sucrose SRC (SUCSRC) linearly decreased with the increasing starch addition in the recombined flour of the three cultivars (Figures 1G,H), while Na_2_CO_3_ SRC (SODSRC) increased with the increasing addition of starch (Figure 1I). The highest LASRC and SUCSRC were found in ZM9023 and the lowest in NM13. Water SRC (WSRC) first increased and then decreased with increasing starch addition (Figure 1J), and it reached the maximum at 15% of starch addition for NM13 and ZM9023, and at 10% of addition for YM 16.

### Pasting and Thermal Properties

NM13 had the highest value of most pasting parameters, with an exception of the pasting temperature, followed by YM 16 and ZM9023 (Table 1). Starch addition increased the peak viscosity, trough viscosity, breakdown, final viscosity, and setback and peak time of the three cultivars. As compared with native flour, the peak viscosity of the recombined flour with 25% starch addition increased by 19.4, 14.9, and 16.3% for NM13, YM16, and ZM9023, respectively. In addition, increasing starch addition decreased pasting temperature in NM 13, while it showed little effect on YM16 and ZM9023.

**TABLE 1:** Effect of additional amount of starch on starch component, pasting, and thermal properties of wheat.

Cultivar	Treatment	Viscosity properties							Thermal properties			
		Peak V/cP	Trough V/cP	Breakdown/cP	Final V/cP	Setback/cP	Peak time/min	Pasting T/°C	To(°C)	Tp(°C)	Tc(°C)	ΔH(J/g)
NM 13	0	3288 ± 5.65f	1803 ± 2.82f	1485 ± 2.82f	3344 ± 8.48f	1541 ± 5.65f	6.50 ± 0.04c	68.45 ± 0.00a	60.58 ± 0.29a	66.02 ± 0.11a	71.15 ± 0.21a	7.76 ± 0.31e
	5%	3432 ± 2.82e	1911 ± 7.07e	1521 ± 4.24e	3540 ± 7.07e	1629 ± 0.00e	6.60 ± 0.00b	68.38 ± 0.03a	60.59 ± 0.29a	65.84 ± 0.15b	70.99 ± 0.31ab	7.88 ± 0.06de
	10%	3552 ± 14.84d	2013 ± 5.65d	1539 ± 9.19d	3682 ± 5.65d	1669 ± 0.00d	6.60 ± 0.00b	68.10 ± 0.07b	58.38 ± 0.17b	66.00 ± 0.26a	71.20 ± 0.31a	8.23 ± 0.16d
	15%	3600 ± 9.89c	2031 ± 8.48c	1569 ± 1.41c	3730 ± 5.65c	1699 ± 2.82c	6.60 ± 0.00b	67.40 ± 0.07c	57.72 ± 0.14c	65.90 ± 0.26ab	71.13 ± 0.21a	9.51 ± 0.11c
	20%	3758 ± 5.65b	2163 ± 5.65b	1595 ± 0.00b	3870 ± 4.24b	1707 ± 1.41b	6.67 ± 0.00a	67.43 ± 0.03c	56.10 ± 0.25d	66.01 ± 0.15a	70.76 ± 0.39b	10.32 ± 0.39b
	25%	3926 ± 4.94a	2256 ± 6.36a	1670 ± 1.41a	3969 ± 3.53a	1713 ± 2.82a	6.66 ± 0.00a	66.40 ± 0.00d	55.12 ± 0.16e	66.00 ± 0.15a	71.13 ± 0.23a	11.09 ± 0.34a
YM 16	0	3019 ± 3.53f	1688 ± 9.19f	1331 ± 5.65f	3038 ± 7.77f	1350 ± 1.41d	6.27 ± 0.00c	68.90 ± 0.07a	61.55 ± 0.35a	66.00 ± 0.21a	70.31 ± 0.28a	6.59 ± 0.27f
	5%	3131 ± 2.82e	1721 ± 9.89e	1410 ± 7.07e	3101 ± 2.12e	1380 ± 12.02d	6.33 ± 0.00b	68.75 ± 0.07b	61.33 ± 0.31a	65.79 ± 0.11a	70.67 ± 0.37a	7.09 ± 0.34e
	10%	3248 ± 6.36d	1779 ± 7.77d	1469 ± 1.41d	3240 ± 3.53d	1461 ± 4.24c	6.33 ± 0.00b	68.18 ± 0.03c	59.88 ± 0.36b	65.80 ± 0.15a	70.46 ± 0.12a	8.09 ± 0.16d
	15%	3351 ± 7.77c	1843 ± 18.38c	1508 ± 10.61c	3339 ± 4.24c	1496 ± 22.62bc	6.33 ± 0.00b	68.15 ± 0.00cd	58.80 ± 0.25c	65.17 ± 0.14b	70.26 ± 0.01a	9.21 ± 0.39c
	20%	3411 ± 7.07b	1887 ± 14.14b	1524 ± 7.07b	3416 ± 7.77b	1529 ± 21.92b	6.46 ± 0.01a	68.10 ± 0.07cd	57.50 ± 0.38d	66.00 ± 0.18a	70.66 ± 0.34a	10.09 ± 0.11b
	25%	3470 ± 4.24a	1915 ± 8.48a	1555 ± 4.24a	3520 ± 16.26a	1605 ± 24.74a	6.47 ± 0.00a	68.03 ± 0.02d	56.00 ± 0.34e	65.20 ± 0.22b	70.82 ± 0.34a	10.89 ± 0.34a
ZM 9023	0	2040 ± 6.33f	1238 ± 9.19e	802 ± 2.82e	2409 ± 4.24f	1170 ± 4.94f	6.07 ± 0.00d	68.75 ± 0.00bc	63.50 ± 0.21a	65.33 ± 0.29a	69.87 ± 0.31a	5.55 ± 0.11e
	5%	2088 ± 9.19e	1239 ± 5.65e	849 ± 3.53d	2425 ± 9.19e	1186 ± 3.53e	6.20 ± 0.00c	69.22 ± 0.67ab	63.33 ± 0.47a	65.32 ± 0.34a	69.80 ± 0.31a	5.61 ± 0.26e
	10%	2162 ± 2.82d	1285 ± 4.24d	877 ± 1.41c	2486 ± 6.36d	1201 ± 2.12d	6.23 ± 0.04c	68.55 ± 0.21bc	62.33 ± 0.45b	64.96 ± 0.31a	70.00 ± 0.41a	7.32 ± 0.28d
	15%	2217 ± 8.48c	1320 ± 4.24c	897 ± 4.24b	2538 ± 3.53c	1218 ± 0.71c	6.30 ± 0.04b	69.68 ± 0.03a	60.50 ± 0.68c	65.00 ± 0.23a	69.80 ± 0.52a	8.16 ± 0.19c
	20%	2339 ± 7.77b	1403 ± 8.48b	936 ± 0.71a	2639 ± 9.89b	1236 ± 1.41b	6.40 ± 0.00a	68.32 ± 0.03c	60.23 ± 0.23c	65.29 ± 0.53a	70.02 ± 0.43a	9.08 ± 0.15b
	25%	2373 ± 7.77a	1430 ± 4.94a	944 ± 2.82a	2705 ± 7.77a	1276 ± 2.82a	6.40 ± 0.00a	66.47 ± 0.03d	58.90 ± 0.21d	65.30 ± 0.29a	69.88 ± 0.29a	10.00 ± 0.33a

*NM13, YM16, and ZM9023 indicate Ningmai 13, Yangmai 16 and Zhengmai 9023, respectively. Peak V means peak viscosity, Trough V means trough viscosity, Final V means final viscosity, Pasting T means pasting temperature, To, Tp, Tc, and ΔH represent the onset temperature, peak temperature, conclusion temperature, and enthalpy, Softening D means softening degree, FQN means Farinograph quality number. Data are means of three replicates. Different small letters in the same column with the same cultivar are significantly different at the 0.05 probability level.*

NM13 had the highest value of Tp, Tc, and ΔH, followed by YM16 and ZM9023, while ZM9023 had the highest To value (Table 1). Starch addition decreased the value of To and increased the value of ΔH of the three cultivars, while it had little effect on the value of Tp and Tc. As compared with native flour, the To value of the recombined flour with 25% starch addition decreased by 9.01, 9.02, and 7.24%, respectively while the ΔH of the recombined flour increased by 42.91, 65.25, and 32.46%, respectively.

### Dough Rheology and Texture Profile

The addition of starch dramatically decreased dough development time, stability time, farinograph quality number (FQN), cohesiveness, and adhesiveness, while it increased softening degree in all the three cultivars (Table 2). However, starch addition had no significant influence on water absorption in ZM9023, while it slightly increased in NM13 and YM16 when starch addition was less than 15%. ZM9023 had the highest values of dough development time, stability time, FQN, cohesiveness, and adhesiveness, and the lowest softening degree among the three cultivars (Table 2). It was contrary for NM13 when compared to ZM9023.

**TABLE 2:** Effect of additional amount of starch on dough rheology of wheat.

Cultivar	Treatment	Farinograph properties				Dough texture properties	
		Stability time/min	Softening D/FU	FQN	Absorption/%	Cohesiveness (g)	Adhesiveness (g⋅sec)
NM13	0	3.00 ± 0.14a	77.5 ± 6.36d	39.5 ± 2.12a	0.632 ± 0.00d	31.94 ± 0.58a	5.01 ± 0.09a
	5%	2.75 ± 0.07b	89.5 ± 3.53d	35.5 ± 0.71b	0.644 ± 0.00c	30.26 ± 0.52b	4.79 ± 0.18a
	10%	2.65 ± 0.07bc	106 ± 1.41c	32.0 ± 1.41c	0.645 ± 0.00bc	29.45 ± 0.91bc	4.06 ± 0.18b
	15%	2.45 ± 0.07cd	118 ± 2.82c	27.5 ± 0.71d	0.6465 ± 0.00abc	28.67 ± 0.97c	4.12 ± 0.26b
	20%	2.30 ± 0.14de	132 ± 4.24b	24.5 ± 0.71e	0.647 ± 0.00ab	26.34 ± 0.32d	3.9 ± 0.11bc
	25%	2.20 ± 0.14e	149 ± 5.65a	21.0 ± 1.41f	0.6485 ± 0.00a	26.02 ± 2.41d	3.77 ± 0.23c
YM16	0	4.80 ± 0.14a	54.0 ± 4.24f	58.5 ± 2.12a	0.634 ± 0.00c	35.14 ± 0.61a	6.01 ± 0.07a
	5%	4.55 ± 0.07a	67.0 ± 2.82e	50.5 ± 0.71b	0.648 ± 0.00ab	33.66 ± 0.34b	5.52 ± 0.14b
	10%	4.25 ± 0.07b	84.0 ± 4.24d	45.0 ± 1.41c	0.6495 ± 0.00a	31.04 ± 0.75c	4.98 ± 0.17c
	15%	3.95 ± 0.07c	100 ± 1.41c	39.5 ± 2.12d	0.645 ± 0.00b	29.83 ± 0.53d	4.22 ± 0.29d
	20%	3.75 ± 0.07cd	110 ± 2.12b	35.5 ± 2.12de	0.647 ± 0.00ab	28.4 ± 0.73e	4.05 ± 0.23de
	25%	3.60 ± 0.14d	120 ± 2.12a	31.5 ± 2.12e	0.645 ± 0.00b	28.02 ± 0.38e	3.98 ± 0.11e
ZM9023	0	8.40 ± 0.42a	26.5 ± 2.12f	106 ± 0.00a	0.645 ± 0.00a	40.63 ± 0.37a	7.64 ± 0.09a
	5%	8.15 ± 0.21ab	33.5 ± 0.71e	99.5 ± 0.71b	0.649 ± 0.00a	38.58 ± 0.43b	7.08 ± 0.18b
	10%	8.00 ± 0.14ab	41.5 ± 0.71d	94.0 ± 1.41c	0.6485 ± 0.00a	36.39 ± 0.31c	6.46 ± 0.06c
	15%	7.60 ± 0.14bc	46.5 ± 0.71c	89.5 ± 0.71c	0.6485 ± 0.00a	34.59 ± 0.26d	5.64 ± 0.12d
	20%	7.25 ± 0.07cd	51.5 ± 2.12b	80.5 ± 2.12d	0.6485 ± 0.00a	33.49 ± 0.31e	5.26 ± 0.19e
	25%	6.85 ± 0.35d	57.5 ± 2.12a	76.0 ± 2.82d	0.648 ± 0.00a	32.93 ± 0.39f	5.03 ± 0.15f

*NM13, YM16, and ZM9023 indicate Ningmai 13, Yangmai 16 and Zhengmai 9023, respectively. Softening D means softening degree, FQN means Farinograph quality number. Data are means of three replicates. Different small letters in the same column with the same cultivar are significantly different at the 0.05 probability level.*

### Biscuit Properties

**FIGURE 2:**
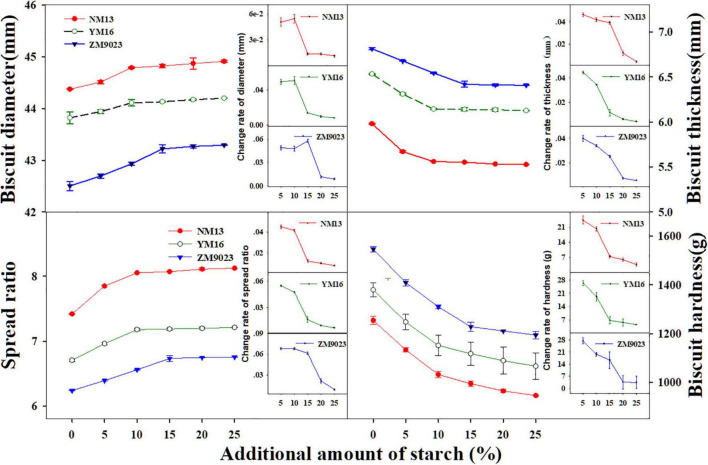
Effect of the addition amount of starch on diameter, thickness, spread ratio, and hardness of short biscuits. NM13, YM16, and ZM9023 indicate Ningmai 13, Yangmai 16 and Zhengmai 9023, respectively. Small figures in the corner of big Figs are rate of change of biscuit traits.

The lowest protein content cultivar, NM 13, showed the largest biscuit diameter and spread ratio, while the highest protein content cultivar ZM9023 showed the lowest biscuit diameter and spread ratio (Figure 2). Biscuit thickness and hardness showed opposite patterns to diameter and spread ratio among the three cultivars. The starch addition increased biscuit diameter and spread ratio, while it decreased biscuit thickness and hardness. Biscuit diameter and spread ratio increased very fast with starch addition at a rate lower than 10% for NM 13 and YM 16, 15% for ZM 9023, and with further starch addition the increase rate leveled off (Figure 2). Similarly, biscuit thickness and hardness decreased rapidly at a starch addition rate lower than 10% for NM 13 and YM 16, and 15% for ZM9023 (Figure 2). The turning point of the change rate of diameter, spread ratio, thickness, and hardness was at 10% of starch addition for NM 13 and YM 16, and 15% for ZM 9023, the corresponding protein content was 9.70, 10.22, and 10.37%.

The lightness (L*) of biscuit and dough was the lowest for NM13 and the highest for ZM9023 (Table 3). The addition of starch gradually increased the values of L* of biscuit along with the addition amount for all cultivars, whereas it decreased the redness (a*) and yellowness (b*) of the biscuit and the dough. With too much starch addition, the short biscuits were light in color and undesirable. Starch addition increased scores of biscuit appearance, which reached a maximum at the starch addition rate of ca. 10–15% (Table 3). But there were some cracks that appeared in the biscuit when starch addition amount exceeded 10% for NM13 and YM16, and 15% for ZM9023. The sensory evaluation score increased faster at starch addition from 0 to 10% than from 15 to 25%.

**TABLE 3:** Effect of additional amount of starch on the sensory evaluation, colors, and cracks on the biscuit.

Cultivar	Treatment	Sensory evaluation							Biscuit colors			IABSB	
		Appearance	Clearness	Gumminess	Crispness	Mouth feel	Texture	Total score	L*	a*	b*	CA (mm2)	RCA(%)
NM13	0	9.67 ± 0.06c	10.00 ± 0.00a	9.67 ± 0.14c	43.5 ± 0.86b	8.83 ± 0.28c	10.00 ± 0.00a	91.66 ± 0.84c	65.6 ± 0.24d	5.68 ± 1.21a	28.08 ± 1.61a	0.50 ± 0.01c	52.05 ± 1.19a
	5%	9.73 ± 0.06bc	10.00 ± 0.00a	9.75 ± 0.00bc	44.0 ± 0.86ab	9.00 ± 0.00c	10.00 ± 0.00a	92.48 ± 0.89b	66.22 ± 0.19d	4.46 ± 1.56a	25.31 ± 0.12ab	0.51 ± 0.01bc	51.64 ± 0.96ab
	10%	9.97 ± 0.06a	10.00 ± 0.00a	9.83 ± 0.14abc	44.6 ± 0.287a	9.67 ± 0.28b	10.00 ± 0.00a	94.13 ± 0.34a	68.99 ± 1.73c	3.67 ± 0.16a	25.25 ± 2.78ab	0.50 ± 0.02c	50.74 ± 0.46abc
	15%	9.87 ± 0.15ab	10.00 ± 0.00a	9.83 ± 0.14abc	44.8 ± 0.283a	10.00 ± 0.00a	10.00 ± 0.00a	94.53 ± 0.11a	71.15 ± 0.01b	3.66 ± 0.13a	24.12 ± 0.55ab	0.55 ± 0.02a	48.67 ± 2.22c
	20%	9.77 ± 0.06bc	10.00 ± 0.00a	9.92 ± 0.14ab	44.8 ± 0.283a	10.00 ± 0.00a	10.00 ± 0.00a	94.51 ± 0.25a	71.32 ± 0.05b	3.45 ± 0.19a	24.52 ± 0.58ab	0.55 ± 0.02ab	49.44 ± 1.21bc
	25%	9.67 ± 0.06c	10.00 ± 0.00a	10.00 ± 0.00a	44.8 ± 0.283a	10.00 ± 0.00a	10.00 ± 0.00a	94.5 ± 0.26a	74.19 ± 0.09a	3.42 ± 0.52a	22.28 ± 1.58b	0.54 ± 0.02abc	48.95 ± 0.95c
YM16	0	9.63 ± 0.06b	10.00 ± 0.00a	9.58 ± 0.14c	43.0 ± 0.00b	8.67 ± 0.28d	10.00 ± 0.00a	90.88 ± 0.21d	69.71 ± 0.21d	4.88 ± 0.78a	25.59 ± 0.94a	0.46 ± 0.00c	56.6 ± 4.72a
	5%	9.73 ± 0.06ab	10.00 ± 0.00a	9.67 ± 0.14bc	43.5 ± 0.86b	9.00 ± 0.00c	10.00 ± 0.00a	91.9 ± 0.77c	70.02 ± 0.14d	4.67 ± 0.11a	24.61 ± 0.71ab	0.46 ± 0.02c	56.48 ± 0.68a
	10%	9.83 ± 0.06a	10.00 ± 0.00a	9.83 ± 0.14ab	44.5 ± 0.00a	9.50 ± 0.00b	10.00 ± 0.00a	93.66 ± 0.21b	72.56 ± 0.02c	4.44 ± 0.55ab	24.27 ± 0.35bc	0.48 ± 0.02c	53.57 ± 0.93a
	15%	9.77 ± 0.11a	10.00 ± 0.00a	9.92 ± 0.14a	44.6 ± 0.287a	9.83 ± 0.28a	10.00 ± 0.00a	94.18 ± 0.02ab	72.86 ± 0.47c	3.99 ± 0.13ab	23.73 ± 1.08bc	0.55 ± 0.01b	52.05 ± 0.46a
	20%	9.73 ± 0.06ab	10.00 ± 0.00a	9.92 ± 0.14a	44.8 ± 0.283a	10.00 ± 0.00a	10.00 ± 0.00a	94.48 ± 0.25a	74.42 ± 0.45b	3.42 ± 0.46ab	23.43 ± 0.81c	0.58 ± 0.01a	54.96 ± 4.14a
	25%	9.63 ± 0.06b	10.00 ± 0.00a	10.00 ± 0.00a	44.8 ± 0.283a	10.00 ± 0.00a	10.00 ± 0.00a	94.46 ± 0.23a	76.7 ± 0.38a	3.16 ± 0.96b	22.4 ± 1.41d	0.57 ± 0.00ab	53.24 ± 1.68a
ZM9023	0	9.6 ± 0.00c	10.00 ± 0.00a	9.5 ± 0.00d	43.0 ± 0.00b	8.67 ± 0.28c	10.00 ± 0.00a	90.76 ± 0.28d	71.25 ± 0.25a	5.93 ± 3.69a	26.67 ± 0.79a	0.45 ± 0.00c	58.34 ± 0.64a
	5%	9.67 ± 0.06bc	10.00 ± 0.00a	9.67 ± 0.14cd	43.5 ± 0.86ab	8.83 ± 0.28bc	10.00 ± 0.00a	91.66 ± 0.71c	71.91 ± 0.77de	5.97 ± 4.07a	26.07 ± 0.85a	0.46 ± 0.01c	56.87 ± 0.76ab
	10%	9.73 ± 0.06ab	10.00 ± 0.00a	9.75 ± 0.00bc	43.5 ± 0.86ab	9.33 ± 0.57b	10.00 ± 0.00a	92.31 ± 0.71b	72.53 ± 0.01cd	3.03 ± 0.77ab	23.38 ± 0.02b	0.48 ± 0.01b	56.95 ± 0.85ab
	15%	9.8 ± 0.00a	10.00 ± 0.00a	9.83 ± 0.14abc	44.5 ± 0.00a	10.00 ± 0.00a	10.00 ± 0.00a	94.13 ± 0.14a	73.43 ± 0.43bc	3.03 ± 0.77ab	23.38 ± 0.02b	0.51 ± 0.01a	57.04 ± 1.47ab
	20%	9.77 ± 0.06a	10.00 ± 0.00a	9.92 ± 0.14ab	44.5 ± 0.00a	10.00 ± 0.00a	10.00 ± 0.00a	94.18 ± 0.12a	74.43 ± 0.43b	1.55 ± 0.15ab	22.49 ± 0.64b	0.51 ± 0.02a	54.58 ± 2.14bc
	25%	9.63 ± 0.06c	10.00 ± 0.00a	10.00 ± 0.00a	44.5 ± 0.00a	10.00 ± 0.00a	10.00 ± 0.00a	94.13 ± 0.05a	76.49 ± 0.29a	0.77 ± 0.17b	20.89 ± 0.06c	0.51 ± 0.01a	53.01 ± 0.44c

*NM13, YM16, and ZM9023 indicate Ningmai 13, Yangmai 16 and Zhengmai 9023, respectively. IABSB means image analysis of bottom side of biscuit, CA is mean cell area, RCA is ratio of cell to total area. Data are means of three replicates. Different small letters in the same column with the same cultivar are significantly different at the 0.05 probability level.*

**FIGURE 3:**
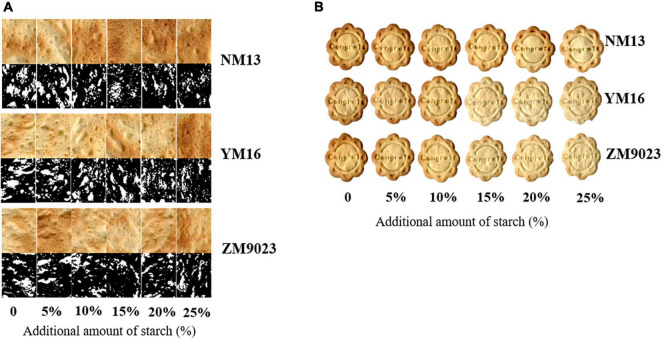
Appearance of short biscuits **(A)** and image of the bottom side of biscuit **(B)**. NM13, YM16, and ZM9023 indicate Ningmai 13, Yangmai 16, and Zhengmai 9023, respectively. In left figure **(B)**, the first, third and fifth lines were view of bottom side of biscuit, and second, fourth and sixth lines were binary images of biscuit.

Image analysis of the biscuits indicated the effect of starch on texture and appearance (Figure 3). The mean cell area increased first and reached the maximum at 20% starch addition and then decreased (Table 3). The ratio of cell to total area showed a decreased trend with the addition of starch in three cultivars. Crumbs of biscuit baked with higher starch content had larger pores but with a lower ratio of cell to total area in the bottom side.

### Relationship of Biscuit Quality With SRC, Pasting, and Thermal Properties and Dough Rheology

Sucrose SRC was significantly negatively correlated with peak viscosity, trough viscosity, breakdown, final viscosity, setback, peak time, Tc and ΔH, while positively with To and pasting temperature (Table 4, *r* = −0.775, −0.83, −0.705, −0.832, −0.823, −0.898, −0.661, −0.845, 0.911, 0.713, respectively, *p* ≤ 0.01). SODSRC was significantly positively related with final viscosity, setback, peak T, and ΔH, while negatively with To and pasting temperature (*r* = 0.504, 0.475, 0.657, 0.8838, −0.817, −0.817, respectively, *p* ≤ 0.05). Consistent with SUCSRC, dough stability time, FQN, cohesiveness, and adhesiveness were significantly negatively correlated with peak viscosity, trough viscosity, breakdown, final viscosity, setback, and peak time, while positively with pasting temperature (Table 4, *p* ≤ 0.01). The softening degree was on the contrary to stability time and FQN.

**TABLE 4:** Linear correlation coefficients between SRC, farinograph, dough texture profile, pasting, and thermal properties.

	Peak V	Trough V	Breakdown	Final V	Setback	Peak T	Pasting T	To	Tp	Tc	ΔH
WSRC	−0.439	−0.4645	−0.406	−0.483*	−0.507*	−0.563*	0.359	0.201	−0.586*	−0.619**	−0.101
SUCSRC	−0.775**	−0.830**	−0.705**	−0.832**	−0.823**	−0.898**	0.713**	0.911**	−0.400	−0.661**	−0.845**
SODSRC	0.448	0.516*	0.370	0.504*	0.475*	0.657**	-0.817**	-0.817**	0.106	0.286	0.884**
LASRC	−0.408	−0.466	−0.341	−0.457	−0.437	−0.574*	0.728**	0.869**	−0.009	−0.216	−0.941**
Stability time	−0.987**	−0.977**	−0.982**	−0.981**	−0.973**	−0.848**	0.480*	0.723**	−0.766**	−0.933**	−0.553*
Softening D	0.934**	0.956**	0.896**	0.957**	0.945**	0.857**	−0.648**	−0.926**	0.553*	0.801**	0.798**
FQN	−0.991**	−0.980**	−0.987**	−0.979**	−0.964**	−0.845**	0.525*	0.796**	−0.703**	−0.896**	−0.652**
Absorption	−0.193	−0.146	−0.2393	−0.148	−0.149	−0.041	−0.186	−0.151	−0.389	−0.307	0.235
Cohesiveness	−0.901**	−0.918**	−0.869**	−0.919**	−0.907**	−0.894**	0.640**	0.937**	−0.510*	−0.756**	−0.865**
Adhesiveness	−0.859**	−0.871**	−0.832**	−0.873**	−0.865**	−0.871**	0.623**	0.924**	−0.455	−0.722**	−0.874**

Peak V means peak viscosity, Trough V means Trough viscosity, Peak T means Peak Time, Pasting T means Pasting temperature, To, Tp, Tc, and ΔH represent the onset temperature, peak temperature, conclusion temperature, and enthalpy; WSRC means water SRC, SUCSRC means sucrose SRC, LASRC means Lactic acid SRC, SODSRC means Na_2_CO_3_ SRC, Softening D means softening degree, FQN means Farinograph. * and ** indicate significance at the levels of 0.05 and 0.01, respectively.

Hardness and thickness of biscuit significantly positively correlated with flour protein, gluten, GMP, and SUCSRC, but negatively related with amylopectin and SODSRC (Table 5). Diameter, spread ratio, and sensory scores of biscuit showed significantly negative correlation with protein, gluten GMP, SUCSRC, and WSRC, but a positive relation with amylopectin and SODSRC. There was no significant correlation between LASRC and thickness, diameter and spread ratio of biscuit.

**TABLE 5:** Linear correlation coefficients between mixed flour characteristics and quality attributes of baking biscuit.

	Quality traits	Hardness	Thickness	Diameter	Spread ratio	Sensory scores
Flour	Protein	0.924**	0.711**	−0.702**	−0.701**	-0.889**
	Gluten	0.938**	0.775**	−0.764**	−0.766**	-0.842**
	GMP	0.927**	0.713**	−0.737**	−0.707**	-0.869**
	Amylopectin	−0.855**	−0.755**	0.612**	0.739**	0.814**
	Amylose	−0.607**	−0.200	0.193	0.183	0.849**
SRC	WSRC	0.189	0.517*	−0.484*	−0.528*	0.190
	SUSRC	0.942**	0.869**	−0.811**	−0.863**	-0.747**
	SODSRC	−0.793**	−0.554*	0.489*	0.540*	0.826**
	LASRC	0.746**	0.435	−0.400	−0.42	-0.857**
Pasting	Peak V	−0.8137**	−0.8772**	0.975**	0.893**	0.446
Properties	Trough V	−0.8468**	−0.9089**	0.9775**	0.9241**	0.4910*
	Breakdown	−0.7662**	−0.8300**	0.9558**	0.8461**	0.393
	Final V	−0.8538**	−0.9288**	0.9812**	0.9417**	0.4981*
	Setback	−0.8530**	−0.9477**	0.9729**	0.9567**	0.5029*
	Peak T	−0.8957**	−0.9514**	0.8969**	0.9451**	0.6165**
	Pasting T	0.6501**	0.5643*	−0.5136*	−0.5632*	-0.5623*
Thermal	To	0.9207**	0.7520**	−0.7702**	−0.7517**	-0.8324**
Properties	Tp	−0.4089	−0.6426**	0.7367**	0.6702**	0.0022
	Tc	−0.6891**	−0.8718**	0.9165**	0.8830**	0.2685
	ΔH	−0.8638**	−0.6122**	0.6110**	0.5993**	0.8836**
Farinographic	Stability time	0.7894**	0.8998**	−0.9855**	-0.9132**	-0.3981
Properties	Softening D	−0.9219**	−0.8827**	0.9124**	0.8896**	0.6746**
	FQN	0.8431**	0.8789**	−0.9780**	-0.8897**	-0.4998*
	Absorption	−0.2309	0.0231	−0.1929	-0.0484	0.4927*
Dough texture properties	Cohesiveness	0.9661**	0.8766**	−0.9113**	-0.8738**	-0.7619**
	Adhesiveness	0.9593**	0.8420**	−0.8835**	−0.8356**	−0.8031**

WSRC means water SRC, SUCSRC means sucrose SRC, LASRC means Lactic acid SRC, SODSRC means Na_2_CO_3_ SRC, Peak V means peak viscosity, Trough V means Trough viscosity, Peak T means Peak Time, Pasting T means Pasting temperature, To, Tp, Tc, and ΔH represent the onset temperature, peak temperature, conclusion temperature, and enthalpy, Softening D means softening degree, FQN means Farinograph quality number. * and ** indicate significance at the levels of 0.05 and 0.01, respectively.

Pasting and thermal properties were closely associated with biscuit quality. Peak viscosity, trough viscosity, breakdown, final viscosity, setback, peak time, Tp, Tc, and ΔH were significantly positively correlated to diameter, spread ratio, and sensory score of biscuit, but negatively correlated to hardness and thickness of the biscuit (Table 5). As for dough rheological and texture properties, stability time, FQN, cohesiveness, and adhesiveness showed strong significant positive correlation with the hardness and thickness of biscuit, and negative with diameter and spread ratio (*p* < 0.01). Water absorption had no relation with hardness, thickness, diameter, and spread ratio.

**FIGURE 4:**
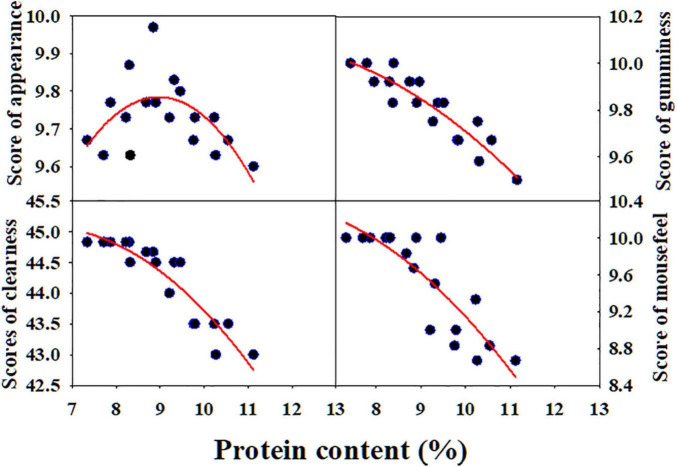
Correlation of protein content with sensory scores of short biscuit. NM13, YM16, and ZM9023 indicate Ningmai 13, Yangmai 16, and Zhengmai 9023, respectively.

Protein content was closely related to biscuit sensory score (Figure 4). There was a quadratic non-linear relationship between protein content and appearance score. When the appearance score reached the highest value, the corresponding protein content was about 9%. Protein content was negatively correlated with the scores of gumminess, clearness of patter, and mouthfeel of biscuit.

## Discussion

Wheat flour with low content of protein and gluten is believed to be an ideal material for biscuits, cookies, and other baking foods (Manley, 2011). Starch is widely applied in food industry due to its unique chemical characteristics of gelling, thickening, and stabilization (Gerits et al., 2015), which provides the possibility to produce flour for biscuit baking by starch addition. In the present study, we add starch to flours of different wheat cultivars differing in grain protein content to produce recombined flour. We observed that the addition of starch improved biscuit-baking quality, as exemplified with the improved diameter and spread ratio of biscuit, and with reduced biscuit thickness and hardness due to the increased addition of starch (Figure 3).

Protein and gluten contents are important factors affecting biscuit-baking quality. Gluten is an essential structure-building protein, which is hydrated to form gluten networks providing viscoelasticity of dough (Lindsay and Skerritt, 1999). Dough development time and stability time are indicators of dough strength, and longer development time and stability time indicates better viscoelastic properties (Panghal et al., 2019). Plenty of gluten networks are necessary for bread making, but it is undesired for biscuit making since gluten network tends to increase biscuit hardness. Protein level, especially when exceeds 10 g/100 g, profoundly affects biscuit hardness and dimensions (Pauly et al., 2013). Our results also showed that protein content was positively related to hardness and thickness of biscuit, which was agreed with other reports (Pauly et al., 2013; Ma and Baik, 2018). Here, contents of protein, gluten and GMP, dough stability time, cohesiveness, and adhesiveness declined rapidly with increasing starch addition (Figure 1 and Table 2). The decrease in protein content and dough strength should be related to the filling of starch granules, resulting in poor gluten network. At an addition rate of 10%, contents of protein of NM13 in the recombined flour of the three cultivars decreased to 9.7%, which may well fit the requirement for biscuit making.

A good quality biscuit is expected to have both a desirable appearance and a tender crumb texture (Ma and Baik, 2018). Our study showed that protein content is quadratic non-linearly related with the appearance of the biscuit (Figure 4). Although less gluten network formation is required in biscuit baking, gluten formation is still very critical in influencing the volume, texture, and appearance of the final baking product. Lack of gluten often gives biscuits of lower quality, both in terms of technological properties and sensory quality (Di Cairano et al., 2018). Here, when starch addition higher than 10 to 15% it caused some cracks on the biscuit surface, which decreased the scores of the appearance of the biscuit. Ma and Baik (2018) evaluated quality characteristics of 15 soft wheat varieties in United States and found that protein content of 7.9–9.7% was suitable for making desirable quality biscuits (Ma and Baik, 2018).

Since gluten free biscuit can be produced, starch may play a more important role in biscuit baking. Viscosity indicates the propensity of starch to gelation, and high starch content in wheat flour is responsible for high peak and final viscosity (Panghal et al., 2019). Starch gelatinization contributes to the biscuit matrix formation (Pauly et al., 2013). Our study showed that most starch pasting and thermal parameters (except for pasting temperature) were positively related to diameter, spread ratio, and sensory scores of biscuit, while negatively related with hardness and thickness of biscuit (Table 5). The starch weak network is formed after the swelling of granules and leaching of starch chains during heat treatment. The short-dough biscuit can be described as a matrix of starch in which gas bubbles of various sizes and shapes were incorporated (Baldino et al., 2014; Sozer et al., 2014). Good biscuits facilitate expansion with a weak functional network formation (Laguna et al., 2011). A firm crumb is an undesirable quality for biscuits (Ma and Baik, 2018). Crumbs of biscuit baked with higher starch content had larger pores, but a lower ratio of cell to total area in the bottom side (Table 3), which might be related to three-dimensional honeycomb network of starch gel after heating (Hedayati and Niakousari, 2018). Starch addition can improve the pasting of starch and inner fill of starch granule in the gluten network. The enlargement of pores may be beneficial to reduce density and improve the crispness of the biscuit.

Solvent retention capacity can provide useful information for predicting the quality of soft wheat products (Kaur et al., 2014). LASRC is associated with glutenin characteristics, SODSRC is related to levels of damaged starch, SUCSRC is associated with pentosan and gliadin characteristics, and WSRC is influenced by all the flour constituents (Zhang et al., 2018). Here, WSRC, SSRC, and LASRC were negatively correlated to biscuit diameter, spread ratio, and sensory scores. Reversely, SODSRC was positively correlated to biscuit diameter, spread ratio, and sensory scores. Moiraghi et al. (2011) reported a significant negative correlation between SUCSRC/WSRC and spread ratio, which is consistent with our results. Peak viscosity, trough viscosity, breakdown, final viscosity, and setback were significantly negatively correlated with SUCSRC, which might suggest that the presence of gliadin and pentosan caused interference to the swelling and rupture of the starch granule. Final viscosity and setback were significantly positively related with SODSRC, which might be due to the damaged starch granules that tend to be easier to gelatinize. It also showed that sucrose and Na_2_CO_3_ SRC were more important than water and lactic acid SRC in determining biscuit quality.

Meanwhile, the increased rate of diameter and spread and the decreased rate of hardness and thickness vs. starch addition amount showed a turning point at 10% starch addition for NM 13 and YM 16, and at 15% for ZM 9023 (Figure 2). At the turning point, the flour protein content of NM13, YM16, and ZM9023 were 8.84, 9.32, and 9.46%, respectively. Thinking about appearance scores, therefore, a criterion of flour protein content around 9% was recommended for baking high quality short biscuits.

## Conclusion

Weak gluten wheat (with low protein and gluten) is in short supply because of low yield due to low N input. Starch addition is an effective way to produce flour with low protein and gluten content to meet the requirements of biscuit industry. Starch addition decreased the contents of protein, gluten and GMP, lactic acid SRC, sucrose SRC, and onset temperature (To), while it increased most pasting parameters and gelatinization enthalpy (ΔH). Viscosity parameters were significantly negatively correlated with dough stability time, farinograph quality number (FQN), and sucrose SRC. Biscuit quality was greatly improved by the addition of starch, as shown by higher diameter, spread ratio, and sensory score of biscuit, but lower thickness and hardness. Starch gelatinization can contribute to biscuit matrix. Viscosity parameters were negatively correlated to hardness and thickness of biscuit, but positively correlated to diameter, spread ratio, and sensory score of biscuit. Considering the effects of starch addition on the dough rheology and biscuit quality, the recombined flour with around 9% protein content after mixing with starch was more suitable for biscuit baking. The interaction between starch and protein during baking needs further investigation. This study provides guidance for the application of wheat starch in the development of high quality biscuit and discloses how starch addition may regulate the properties of flour and the inter-relationships of flour, dough, and biscuit.

## Data Availability Statement

The original contributions presented in the study are included in the article/supplementary material, further inquiries can be directed to the corresponding author/s.

## Author Contributions

LL analyzed the dataset and prepared the first draft. TY and JY critically reviewed the manuscript. QZ and DJ conceived the project idea. MH and JC helped the isolation of starch. WC and TD reviewed the manuscript. All authors contributed to the article and approved the submitted version.

## Conflict of Interest

The authors declare that the research was conducted in the absence of any commercial or financial relationships that could be construed as a potential conflict of interest.

## Publisher’s Note

All claims expressed in this article are solely those of the authors and do not necessarily represent those of their affiliated organizations, or those of the publisher, the editors and the reviewers. Any product that may be evaluated in this article, or claim that may be made by its manufacturer, is not guaranteed or endorsed by the publisher.
